# A New Strain of Preponderant Amphitriploid *Carassius* Clone Juvenile With Integrated Genomes Partly From White Crucian Carp (*C. auratus cuvieri*) Requires Low Dietary Protein

**DOI:** 10.1155/anu/6356786

**Published:** 2025-01-22

**Authors:** Xing Wang, Dong Han, Junyan Jin, Xiaoming Zhu, Haokun Liu, Zhimin Zhang, Yunxia Yang, Shouqi Xie

**Affiliations:** ^1^State Key Laboratory of Fresh Water Ecology and Biotechnology, Institute of Hydrobiology, Chinese Academy of Sciences, Wuhan, Hubei, China; ^2^College of Advanced Agricultural Sciences, University of Chinese Academy of Sciences, Beijing, China; ^3^Hubei Engineering Research Center for Aquatic Animal Nutrition and Feed, Wuhan, Hubei, China; ^4^Key Laboratory of Breeding Biotechnology and Sustainable Aquaculture, Chinese Academy of Sciences, Wuhan, Hubei, China

**Keywords:** antioxidative capacity, *Carassius*, dietary protein level, growth performance, nutritional components, stress status

## Abstract

This study was carried out to search for the protein requirement of a new strain of preponderant amphitriploid *Carassius* clone, which integrated genomes partly from white crucian carp (*C. auratus cuvieri*). Seven groups of fish (body weight: 9.73 ± 0.03 g) were fed with seven isolipidic and isocarbohydrate diets containing 21.38%, 25.82%, 27.94%, 31.36%, 34.23%, 37.87%, and 40.70% crude protein (P21, P24, P27, P30, P33, P36, and P39), respectively. After 8-week feeding, weight gain rate (WGR) and specific growth rate (SGR) were lower in the P30 group than those in the P39 group, but no difference was found in final body weight (FBW), survival, condition factor (CF), or hepatosomatic index (HSI) between different groups. Increased dietary protein decreased feeding rate (FR) and viscerosomatic index (VSI) while improved feed efficiency (FE). Decreased protein retention efficiency (PRE) and improved activity of liver alanine aminotransferase (ALT) and content of plasma ammonia suggested intensified fish amino acid catabolism in high dietary protein groups. The dietary protein requirement of the new *Carassius* clone was as low as 21.38% for growth. The optimal dietary protein for high FE was 39.62% and should be less than 30.56% to maintain the maximum protein retention. High dietary protein might be harmful to the fish due to the increased contents of liver malondialdehyde (MDA) and plasma cortisol. Dietary protein level altered fish body and muscle flavor substance composition. Low dietary protein could obtain high muscle fatty acid, free amino acid, and lipid accumulation, including whole body and muscle crude lipid, plasma total triglyceride (TG), total cholesterol (TC), high-density lipoprotein cholesterol (HDL-c), and low-density lipoprotein cholesterol (LDL-c). Therefore, the recommended dietary protein for this new *Carassius* clone juvenile should be 21.38%–30.56%.

## 1. Introduction

Nowadays, the aquaculture industry has been growing vigorously, and the yield of aquaculture has reached 94.4 million tons due to the fast development of artificial compound feed [1]. Dietary protein is one of the most important ingredients for its high nutrition and price. Compared to land animals, the fish usually requires higher dietary protein [2]. Dietary protein deficiency will slow down the fish growth, while excessive dietary protein will improve less or no more growth sometimes but also increase nitrogenous excretion and feed cost, leading to environmental loading and waste of resources [3–5]. Furthermore, to maintain the long-term healthy development of feed industry, many sustainable protein sources have been exploited to conserve fishery and food resources [6–9]. But fish growth is normally impaired by the high substitution of traditional proteins by novel proteins, and this dilemma limits their use in aquafeed [10–12]. Due to the feed cost, limited dietary protein resources, and environmental legislation, it is urgent to precisely investigate the dietary protein requirement of fish to obtain optimal production, as well as to achieve paramount profit.

With the application of genetic improvement in aquaculture, various new strains/clones of aquaculture animals have been continuously bred. Many studies have confirmed that dietary protein requirement differed from strains/clones of the same species. The growth responses of rainbow trout (*Oncorhynchus mykiss*) and Nile tilapia (*Oreochromis niloticus*) from different genetic groups were found to be different to varying dietary protein levels [13, 14]. Zhou et al. [15] revealed that the dietary protein requirement of a new strain of juvenile Chinese softshell turtle (*Pelodiscus sinensis*) was lower. The protein requirements were found to be different (36.5% and 30.7%, respectively) in gibel carp CAS Ⅲ (*C. gibelio* var. CAS Ⅲ) (85.20 g) and CAS Ⅴ (*C. gibelio* var. CAS Ⅴ) (72.53 g) [16, 17]. The dietary lysine requirements for optimum protein retention also differed between different rainbow trout strains [18]. These suggested that genetic background could affect dietary protein requirement.

*Carassius* spp. is one of the most important freshwater aquaculture categories in China with the annual production over 2.84 million tons [19]. Recently, the newest clone of gibel carp referred to as “preponderant amphitriploid *Carassius* clone” has been created by Lu, Zhou, and Gui [20], and it performs a faster growth and stronger disease resistance than its female parent, gibel carp CAS Ⅲ (unpublished results). One of its several male parents is white crucian carp (*C. auratus cuvieri*) [20], an omnivorous fish which can also ingest zooplankton and phytoplankton by filter-feeding [21] with low dietary protein requirements (20%–30%) [22–24]. Therefore, whether a big difference of protein requirements between the new strain and gibel carp CAS III should be clarified.

On the other hand, previous studies on dietary protein requirement of fishes mainly focused on growth performance and nitrogen balance, but negative effects on muscle quality and organic health caused by excessive protein were normally ignored. Therefore, the present study was designed to investigate the protein requirement of the preponderant amphitriploid *Carassius* clone and to find nutritional components and stress status under high dietary protein, so as to provide some nutritional basis for aquaculture breeding and exploitation of practical feed.

## 2. Materials and Methods

### 2.1. Ethics Statement

All experimental protocols obeyed the Guiding Principles for Care and Use of Laboratory Animals, and the study was approved by the ethics committee of the Institute of Hydrobiology, Chinese Academy of Sciences (IHB, CAS, Protocol No. 2016-018).

### 2.2. Experimental Diets

Seven isolipidic and isocarbohydrate experimental diets containing 21% (P21), 24% (P24), 27% (P27), 30% (P30), 33% (P33), 36% (P36), and 39% (P39) crude protein were formulated with fish meal and casein as protein sources. The diet formulation and nutrient composition were listed in Table 1. All solid ingredients were dried, superfine grinded, weighed according to the formulated ratios, adequately mixed, and then mixed with fish oil and soybean oil. Finally, the mixture was added with water to increase its humidity to be made into pellets (diameter: 1 mm) by laboratory extruder (SLP-45; Fishery Machinery and Instrument Research Institute, Chinese Academy of Fishery Sciences, Shanghai, China). The pellets were dried at 65°C for 4 h and stored at 4°C.

### 2.3. Fish and Rearing Conditions

About 1000 experimental fish, collected from the Breeding Center of Crucian Carp of the Ministry of Agriculture and Rural Affairs of the People's Republic of China (Liangzi Lake Research Base, the Institute of Hydrobiology, Chinese Academy of Sciences, Wuhan, Hubei, People's Republic of China), were acclimated in an indoor recirculating water system (gross water volume: 30 m^3^) and fed with a commercial diet (103, Tongwei Co., Ltd.) for 2 weeks. Before the growth trial, 420 similar size and apparent healthy fish (body weight: 9.73 ± 0.03 g) after 24 h fasting were randomly selected and equally distributed into 21 cylindrical fiberglass tanks (water volume: 285 L, water depth: 0.58 m), which were randomly (by random function of Excel) divided into seven groups for seven experimental diets. During the 8-week rearing period (from August 26, 2022, to October 21, 2022), fish were fed to apparent satiation at 8:30, 13:30, and 16:30, and the uneaten feed were collected 0.5 h after each meal to obtain factual feed intake. Dead fish were promptly removed and recorded. The water temperature was recorded every morning and afternoon, which ranged within 26.4 ± 5.1°C. The pH value, dissolved oxygen, and ammonia nitrogen concentrations were determined every fortnight. They were controlled at 7.44 ± 0.13, more than 6.08 mg/L, and less than 0.15 mg/L, respectively. Light density was 100–120 lx near the water surface, and residual chlorine was less than 0.02 mg/L.

### 2.4. Sample Collection

After the feeding experiment, fish were imposed on a 24-h fasting, then anesthetized by MS-222 (50 mg/kg; tricaine methanesulfonate, Argent Chemical Laboratories Inc., Redmond, WA, USA), and bulk weighed. Eight fish in each tank were randomly selected, of which two were weighed and stored at −20°C for determining the whole body composition, amino acids, and fatty acid profiles. Three fish were conducted to caudal blood sampling using 2-mL heparinized syringes and then centrifuged at 3000 rpm (850 g) for 15 min at 4°C, and the supernatants were separated and stored at −80°C for the measurement of plasma biochemical parameters. Then these three fish were dissected on ice, and the livers, midguts, and dorsal muscles were immediately detached. The livers and midguts were frozen in liquid nitrogen and stored at −80°C for succedent assay of enzymatic activities. The muscle samples were frozen at −20°C to analyze the muscle composition. After the body lengths were measured, another three fish were weighed and dissected, and their viscera and livers were isolated and weighed, for the calculation of morphological parameters.

### 2.5. Biochemical Analysis

The moisture, crude protein, crude lipid, and ash contents of the feed and whole fish body were measured by the method of AOAC [25]. Moisture content was calculated as the percentage of weight loss before and after drying in an oven at 105°C. The assay of crude protein content (N × 6.25) was based on the Kjeldahl method, using an Auto-Kjeldahl apparatus (Kjeltec-8400, FOSS Tecator, Haganas, Sweden) after samples were dissolved by concentrated sulfuric acid. Crude lipid content was assayed through ether extraction with a Soxtec system (Soxtec System HT Tecator, Extraction Unit, Hoganas, Sweden). Ash content was determined after samples were incinerated a muffle furnace (Muffle furnace, Yingshan, Hubei, China) at 550°C for 12 h. Gross energy was determined by direct combustion in an adiabatic bomb calorimeter (SDC311, Hunan Sundy Science and Technology Development Co., Ltd, Changsha, Hunan, China).

The assay of the fish muscle composition was the same as the methods described above, except for crude lipid. Fish muscle lipid was extracted in the chloroform–methanol solution (V/V = 1:1), then 1.6% CaCl_2_ was mixed into the solution to make it layered, and the upper inorganic phase was subsequently deserted. After drying in an oven at 70°C to constant weight, the lipids were obtained, and the crude lipid content was calculated as a percentage between the weight of the lipid and sample.

The pretreatment of determination of free flavor amino acids of dorsal muscles were preformed according to the method detailed by Xu et al. [26]. Then an amino acid analyzer (A300, MembraPure GmbH, Germany) was used to analyze the contents of different amino acids.

Fatty acid profiles of dorsal muscles were determined according to the description in Fei et al. [27]. Briefly, fatty acids were extracted with the chloroform–methanol solution (V/V = 2:1), afterward reacting with methanolic sulfate to prepare fatty acid methyl esters (FAMEs). FAMEs were separated and analyzed by a gas chromatograph-mass spectrometer (GC-MS, 7890A, Agilent Technologies, USA).

The activities of lipase (A054-2-1) and α-amylase (C016-1-1) in the midguts; alanine aminotransferase (ALT, C009-2-1), aspartate aminotransferase (AST, C010-2-1), and catalase (CAT, A007-1-1) in the livers; and the contents of glucose (GLU, A154-1-1), ammonia (A086-1-1), and cortisol (H094-1-2) in plasma, malondialdehyde (MDA, A003-1-2), and heat shock protein 70 (HSP70, H264-2-2) in the livers were assayed with commercial kits (Nanjing Jiancheng Bioengineering Institute, Nanjing, Jiangsu, China) under the instructions of the manufacturer. The activities of trypsin and chymotrypsin in the midguts were determined according to the method by Yu et al. [28].

Part of plasma biochemical parameters, including the contents of total protein (TP), total triglyceride (TG), total cholesterol (TC), high-density lipoprotein cholesterol (HDL-c), and low-density lipoprotein cholesterol (LDL-c), and the activities of ALT and AST were assayed by a Full Automatic Biochemical Analyzer (Mindray BS-460, Shenzhen, China) using standard kits according to the manufacturer's instructions.

### 2.6. Statistical Analysis

All data are presented as the mean ± SEM. SPSS 23.0 (SPSS Inc., Chicago, IL, USA) was appointed to conduct homogeneity of variance test, one-way analysis of variance (ANOVA), and Duncan's multiple range test to compare means and test whether the differences are significant between treatments, which was considered to exist when *p* < 0.05.

## 3. Results

### 3.1. Growth Performance and Morphological Parameters

Table 2 shows the growth performance and morphological parameters of the preponderant amphitriploid *Carassius* clone fed diets with different protein levels. After 56-day rearing, no significant differences of final body weight (FBW) or survival rate (SR, while weight gain rate (WGR) and specific growth rate (SGR) were lower in the P30 group (*p* < 0.05). Condition factor (CF) and hepatosomatic index (HSI) showed no difference between different dietary proteins (*p* > 0.05). Feeding rate (FR) decreased as dietary protein level increased, and that of the P21 group was significantly higher than other groups (*p* < 0.05). Feed efficiency (FE) increased with dietary protein levels, with the lowest in the P21 group (*p* < 0.05), and the highest in the P39 group, but not significantly different with the P36 group (*p* > 0.05). Protein retention efficiency (PRE) generally decreased with dietary protein levels, with the highest in the P21, P24, and P27 groups (*p* < 0.05) and lower in other four groups (*p* < 0.05). Viscerosomatic index (VSI) was the highest in the P21 group but not significantly different with the P24, P27, P30, and P33 groups (*p* > 0.05), and lowest in the P39 group, but not significantly different with the P27, P30, P33, and P36 groups (*p* > 0.05).

**Figure 1:**
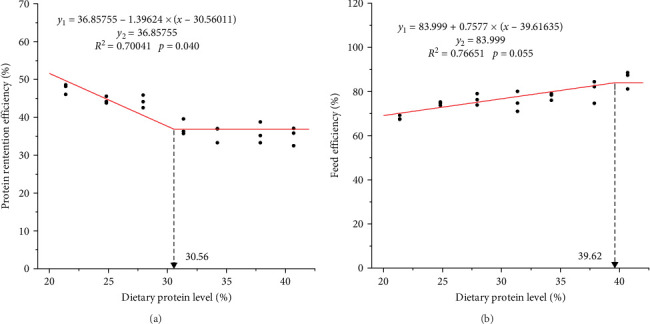
Broken-line regression analysis between (A) protein retention efficiency and (B) feed efficiency and dietary protein level in a preponderant amphitriploid *Carassius* clone.

Broken-line regression analysis showed that the dietary protein requirement for high protein retention should be less than 30.56% (Figure 1A), and the protein requirement for high FE was 39.62% (Figure 1B).

### 3.2. Liver Biochemical Parameters

Table 3 shows the liver biochemical parameters of the fish fed diets with different protein levels. The activities of ALT increased with dietary protein levels, with higher value in the P36 group (*p* < 0.05). The activity of CAT in the P39 group was significantly lower than those in the P21, P24, P27, P30, and P33 groups (*p* < 0.05) and was not significantly different between the P36 group (*p* > 0.05). The contents of MDA were higher in the P36 and P39 groups (*p* < 0.05) but not significantly than those in the P30 and P33 groups (*p* > 0.05). The activities of AST and the contents of HSP70 were not different between groups (*p* > 0.05).

### 3.3. Plasma Biochemical Parameters

Table 4 showes the plasma biochemical parameters of the fish fed diets with different protein levels. The concentrations of TG, TC, HDL-c, and LDL-c decreased as the dietary protein level increased. The concentrations of ammonia and cortisol increased with dietary protein levels, with the highest values obtained in the P36 and P39 groups (*p* < 0.05). However, the contents of the TP and GLU and the activities of ALT or AST showed no significant difference between groups (*p* > 0.05).

### 3.4. Amino Acid Profile

Table 5 shows the sum of free flavor amino acid contents (mg/g in fresh weight) in the muscle of the fish fed diets with different protein levels. The dietary protein content significantly influenced the contents of flavor amino acids (*p* < 0.05). With increasing dietary protein, the sum of umami amino acids (aspartic acid and glutamic acid), sweet amino acids (aspartic acid, glycine, alanine, methionine, leucine, arginine, and proline), and bitter amino acids (valine, isoleucine, leucine, tyrosine, phenylalanine, histidine, and lysine) generally decreased (*p* < 0.05).

### 3.5. Fatty Acid Profile

Table 6 shows the main fatty acid composition (% of total fatty acids) of the fish fed diets with different protein levels. Apparently, dietary treatment markedly affected the contents of C14:0, C17:0, C18:0, C20:0, sum of saturated fatty acids (SFA), C17:1n-7, sum of monounsaturated fatty acids (MUFA), C18:3n-3, C20:5n-3 (EPA), C18:2n-6, C20:2n-6 and C20:3n-6, and sum of n-6 polyunsaturated fatty acids (n-6PUFA) (*p* < 0.05). However, no significant difference was observed in the contents of C15:0, C16:0, C16:1n-7, C18:1n-9, C20:1n-9, C18:3n-6, C20:4n-6 (ARA), C22:2n-6, C22:6n-3 (DHA), sum of n-3 polyunsaturated fatty acids (n-3PUFA), and sum of highly polyunsaturated fatty acids (HUFA) between groups (*p* > 0.05).

### 3.6. Whole Body and Muscle Composition

Table 7 shows the whole body and muscle composition of the fish fed diets with different protein levels. As depicted, the whole body moisture content was higher in the P33, P36, and P39 groups (*p* < 0.05). The whole body crude protein increased with dietary protein levels and was the highest in the P36 group (*p* < 0.05). Contrastively, the whole body crude lipid decreased with dietary protein levels and was the lowest in the P36 group (*p* < 0.05). The whole body ash content was not significantly different between groups (*p* > 0.05).

The muscle moisture content was the lowest in the P21 group (*p* < 0.05) and the highest in the P27, P30, P33, P36, and P39 groups (*p* < 0.05). The muscle crude protein content was the lowest in the P21 group but not significantly than the P24, P27, P30, P33, and P39 groups (*p* > 0.05), except the P36 group (*p* < 0.05). The muscle crude lipid content was the highest in the P21 group (*p* < 0.05) and the lowest in the P36 group, despite not significantly than the P27, P30, P33, and P39 groups (*p* > 0.05).

### 3.7. Intestinal Digestive Enzymatic Activity

Table 8 shows the activities of digestive enzymes in intestine of the fish fed diets with different protein levels. As demonstrated, the activities of trypsin and chymotrypsin increased with dietary protein levels. The activity of the trypsin in the P39 group was significantly higher than those in the P21, P24, and P27 groups (*p* < 0.05). The activity of chymotrypsin in the P39 group was significantly higher than those in the P21, P24, P27, and P30 groups (*p* < 0.05). But no significant differences of the activities of lipase or α-amylase were observed between groups (*p* > 0.05).

## 4. Discussion

The present study found that the growth (WGR and SGR) of a new strain of preponderant amphitriploid *Carassius* clone at lower dietary protein was similar to those at higher dietary protein levels. It was different from most reports that low dietary protein resulted in poor growth [29–31]. The present study found that the increased FR at low dietary protein could help maintain the new fish strain a normal growth by high feed intake when fed low dietary protein, but the feed conversion was still much lower. Similar observation was reported in juvenile dotted gizzard shad (*Konosirus punctatus*) that the fish fed with 22.52% dietary protein grew as well as those fed with 45.78% by increasing feed intake [32]. However, protein requirements of juvenile (3.7 g), preadult (85.2 g), and adult (180.3 g) gibel carp CAS Ⅲ (the female parent of the preponderant amphitriploid *Carassius* clone) were 41.4%, 36.5%, and 36.9%, respectively [16, 33]. An obvious gap of the requirements between the new strain and its female parent might be because the new strain integrated genomes partly from white crucian carp, in which the protein requirement is lower than gibel carp CAS Ⅲ [23]. Likewise, gibel carp CAS Ⅴ had a lower protein requirement than gibel carp CAS Ⅲ [16, 17], because it included a part of the genetic information from blunt snout bream (*Megalobrama amblycephala*), a herbivorous fish requiring low dietary protein [34–36]. The present result illustrated that the protein requirements of the new strain were much associated with their different parents. Extended study should be conducted to compare the ability to utilization of protein between strains and their genetic background.

PRE can characterize nitrogen deposition [37]. High dietary protein which resulted in poor protein retention has been confirmed in many reports [38–40]. According to the broken-line models, the dietary protein level of the new strain should not surpass 30.56%, so as to avoid nitrogenous waste. Overall, the dietary protein content for the preponderant amphitriploid *Carassius* clone was supposed to be ranged in 21.38%–30.56%.

The reduced PRE in high dietary protein groups could be accounted for the activities of liver ALT and the contents of plasma ammonia [41]. In this study, fish liver ALT activity increased with dietary protein levels, and Metón et al. [42] also found that ALT activity in the liver of gilthead sea bream (*Sparus aurata*) was higher when fed with diet containing higher protein. The present study showed no significantly difference liver AST activity among treatments, and the same result was reported in rainbow trout [43]. Alanine and aspartate are both gluconeogenic amino acids, but quantitatively, alanine is the most important, and this might explain why ALT was affected by the dietary treatment but AST was not [44]. According to Cai, Wermerskirchen, and Adelman [45] findings, ammonia excretion could indicate dietary protein adequacy for fish. The current study found that plasma ammonia was extremely high in the fish fed the diet contained excessive protein. Yang, Liou, and Liu [40] reported that the amount of postprandial ammonia excreted by silver perch (*Bidyanus bidyanus*) was positively correlated with dietary protein level. It is pertinent to note that high dietary protein could increase the burden of ammonia excretion in the fish and further cause higher load to the water environment [46].

The present study showed that the new strain of preponderant amphitriploid *Carassius* clone fed with excessive protein might get into a weak antioxidative status. High dietary protein dimmed the activity of CAT and increased the content of MDA in the liver, agreeing with many other reports that the contents of liver MDA was negatively correlated to the CAT activity. It meant that too much dietary protein might weaken the antioxidative status of the fish and led to an accumulation of peroxides [47–49]. Other studies concerning different dietary amino acid, lipid, or carbohydrate levels also suggested that exorbitant nutrients in diet would induce more production of MDA [50–52]. And a study on crayfish (*Procambarus clarkia*) showed that the liver antioxidative capacity was degraded whether dietary protein level was high or low [53]. But the present study revealed that low dietary protein of 21.38% did not show harmful effects based on the measured parameters. The difference illustrated that low dietary protein was enough to maintain a sound antioxidation of the new strain of preponderant amphitriploid *Carassius* clone. Certainly, more evaluations must be conducted as that antioxidative capacity is a systematic and lifelong ability.

HSP70 and cortisol are two major markers of stress response and tolerance [54, 55]. Many studies found that the level of hepatic HSP70 or plasma cortisol was changed by ammonia stress, oxidative stress, and undesirable nutriture [56–58]. In the current study, although the content of HSP70 in the liver was impervious to the level of protein in diet, that of plasma cortisol was higher in high dietary protein groups, particularly in 37.87% and 40.70% protein, virtually in line with the results of plasma ammonia and MDA. On the one hand, the high concentration of plasma ammonia elevated the excretory burden [46], and the mass of MDA elicited the oxidative stress [47]. They combinedly contributed to the high level of cortisol, whereby it can be affirmed that the new strain of preponderant amphitriploid *Carassius* clone would confront with serious stress if its dietary protein was in excess.

Free amino acids and fatty acid composition are two important contributors to fish muscle flavor and nutritional values [59]. The current study found that the contents of free umami amino acids, sweet amino acids, and bitter amino acids of muscle in low dietary protein groups were higher. A study in grass carp (*Ctenopharyngodon idella*) similarly displayed that the free amino acids reached to maximum at moderate dietary protein level [60]. Besides, the contents of C20:4n-6 (ARA), C22:6n-3 (DHA), n-3PUFA, and HUFA were not impacted by protein levels, while low protein diet-fed fish had higher content of EPA, consistent with Dong et al. [61]. And the content of n-6PUFA in fish fed with lowest protein diet was as many as that in fish fed with high protein diets. The present study found that low dietary protein might boost flesh flavor and nutritional values of the new gibel carp clone; however, other flavor substances and texture should be further analyzed.

The results of the whole fish body and dorsal muscle composition showed that high dietary protein increased the contents of crude protein, agreeing with many other studies in European grayling (*Thymallus thymallus*), juvenile giant grouper (*Epinephelus lanceolatus*), and orange-spotted grouper (*E. coioides*) [62–64]. It can be an evidence of which high dietary protein could accelerate the synthesis and deposition rate of body protein [65]. Whole body and muscle lipid contents ascended with dietary protein. It could owe to the fact that low-protein diets promoted feed intake and the attendant increase ingestion of lipid and carbohydrate resulted in high body fat [66]. The present study also showed that dietary protein level did not influence plasma GLU, and the contents of plasma TG, TC, HDL-c, and LDL-c were concomitantly diminished by increased dietary protein and decreased lipid ingestion, which was similar to the study in juvenile obscure puffer (*Takifugu obscurus*) [67]. In the findings of Guo et al. [68], glycolysis and lipid synthesis was upregulated after fish were fed with lower-protein diet, thus leading to no impact on glycemia and a boost on blood fat. Hematological parameters were prevalently used to reflect systemic physiological and metabolic and health status [69]. Xv et al. [70] proved that high blood fat would increase liver damage and elevation of activities of plasma ALT and AST. The present study showed that the activities of plasma ALT and AST were not remarkably changed with the dietary protein level or the blood fat, implying that both high and low protein-contained diets would not affect the liver function of the new strain of preponderant amphitriploid *Carassius* clone, and the insignificant different HSI between groups also confirmed it.

It has been widely reported in many fishes that the diet could markedly alter the activities of digestive enzymes [71–73]. The present study showed that the activities of trypsin and chymotrypsin first improved when dietary protein increased from 21.38% to 31.36% or 34.23% and then remained constant. It is similar to some previous researches at a certain protein level range [74, 75], possibly because higher protein diet brought about more substrate availability, and much more protein led to saturations between enzymes and substrates [66].

## 5. Conclusion

In conclusion, the dietary protein requirement of the new strain of preponderant amphitriploid *Carassius* clone was as low as 21.38% for growth, because the fish could well adapt to a low protein-contained diet via increasing feed intake. On the other hand, the dietary protein requirement for high feed conversion efficiency was 39.62%, and the dietary protein content should be less than 30.56% to maintain the maximum protein retention. Low dietary protein could obtain high muscle fatty acid and free flavor amino acid and low plasma ammonia and cortisol.

## Figures and Tables

**Table 1:** Ingredients and proximate composition of the experimental diets of different protein levels.

Diets	P21	P24	P27	P30	P33	P36	P39
*Ingredients* (*%*)							
Fish meal	15.00	15.00	15.00	15.00	15.00	15.00	15.00
Casein	11.90	15.27	18.65	22.02	25.40	28.78	32.15
Corn starch	37.00	37.00	37.00	37.00	37.00	37.00	37.00
Vitamin premix^a^	0.39	0.39	0.39	0.39	0.39	0.39	0.39
Mineral premix^b^	5.00	5.00	5.00	5.00	5.00	5.00	5.00
Fish oil	3.51	3.50	3.49	3.49	3.48	3.48	3.47
Soybean oil	3.51	3.50	3.49	3.49	3.48	3.48	3.47
Choline chloride	0.11	0.11	0.11	0.11	0.11	0.11	0.11
CMC	2.00	2.00	2.00	2.00	2.00	2.00	2.00
Cellulose	21.59	18.23	14.86	11.50	8.13	4.77	1.40
*Proximate composition*							
Crude protein (%)	21.38	25.82	27.94	31.36	34.23	37.87	40.70
Crude lipid (%)	8.02	8.10	8.02	8.16	8.18	8.16	8.20
Moisture (% wet weight)	1.21	2.93	0.83	4.02	3.04	2.10	4.06
Ash (%)	6.39	6.43	6.59	6.65	6.91	6.90	7.04
Gross energy (kJ/g)	19.72	19.93	20.05	20.37	20.55	20.79	21.00

^a^Vitamin premix (mg·kg^−1^ diet): vitamin B_1_, 20; vitamin B_2_, 20; vitamin B_6_, 20; vitamin B_12_, 0.020; folic acid, 5; calcium pantothenate, 50; inositol, 100; niacin, 100; biotin, 0.1; cellulose, 3412; vitamin C, 100; vitamin A, 11; vitamin D, 2; vitamin E, 50; vitamin K, 10.

^b^Mineral premix (mg·kg^−1^ diet): NaCl, 500.00; MgSO_4_·7H_2_O, 8155.60; NaH_2_PO_4_·2H_2_O, 12,500.00; KH_2_PO_4_, 16,000.00; CaHPO_4_·2H_2_O, 7650.65; FeSO_4_·7H_2_O, 2286.18; C_6_H_10_CaO_6_·5H_2_O, 1750.00; ZnSO_4_·7H_2_O, 177.97; MnSO_4_·H_2_O, 61.40; CuSO_4_·5H_2_O, 15.50; CoSO_4_·7H_2_O, 0.91; KI, 1.50; Na_2_SeO_3_, 0.60; corn starch, 899.69.

**Table 2:** Growth performance and morphological parameters of a preponderant amphitriploid *Carassius* clone fed diets with different protein levels.

Parameters	P21	P24	P27	P30	P33	P36	P39
IBW^1^ (g)	9.77 ± 0.07	9.60 ± 0.07	9.84 ± 0.05	9.68 ± 0.04	9.81 ± 0.08	9.64 ± 0.11	9.75 ± 0.07
FR^2^ (%BW/day)	3.31 ± 0.03^e^	3.02 ± 0.03^d^	2.93 ± 0.03^cd^	2.90 ± 0.07^bcd^	2.84 ± 0.03^bc^	2.78 ± 0.07^b^	2.64 ± 0.05^a^
FBW^3^ (g)	43.22 ± 0.37	42.21 ± 0.57	43.07 ± 0.63	40.23 ± 1.27	41.80 ± 0.51	41.82 ± 1.26	43.37 ± 0.98
WGR^4^ (%)	342.26 ± 0.60^ab^	339.91 ± 7.70^ab^	337.67 ± 9.18^ab^	315.63 ± 11.54^a^	326.15 ± 0.77^ab^	333.83 ± 12.42^ab^	344.84 ± 9.06^b^
SGR^5^ (%/day)	2.65 ± 0.00^ab^	2.64 ± 0.03^ab^	2.64 ± 0.04^ab^	2.54 ± 0.05^a^	2.59 ± 0.00^ab^	2.62 ± 0.05^ab^	2.66 ± 0.04^b^
SR^6^ (%)	100.00 ± 0.00	100.00 ± 0.00	100.00 ± 0.00	98.33 ± 1.67	98.33 ± 1.67	100.00 ± 0.00	100.00 ± 0.00
FE^7^ (%)	68.07 ± 0.58^a^	74.54 ± 0.45^b^	76.42 ± 1.50^b^	75.31 ± 2.63^b^	77.86 ± 0.92^b^	80.47 ± 2.96^bc^	85.75 ± 2.31^c^
PRE^8^ (%)	47.62 ± 0.78^b^	44.52 ± 0.55^b^	44.18 ± 0.96^b^	37.21 ± 1.21^a^	35.75 ± 1.23^a^	35.76 ± 1.61^a^	35.15 ± 1.38^a^
CF^9^ (g/cm^3^)	2.92 ± 0.02	2.87 ± 0.06	2.85 ± 0.05	2.79 ± 0.03	2.81 ± 0.05	2.80 ± 0.05	2.80 ± 0.04
VSI^10^ (%)	11.11 ± 0.61^c^	10.86 ± 0.60^bc^	9.92 ± 0.45^abc^	9.40 ± 0.69^abc^	9.55 ± 0.55^abc^	9.26 ± 0.51^ab^	8.99 ± 0.56^a^
HSI^11^ (%)	3.98 ± 0.27	3.91 ± 0.33	3.67 ± 0.29	3.44 ± 0.20	3.39 ± 0.20	3.52 ± 0.14	3.27 ± 0.11

*Note:* Data are presented as the mean ± SEM (*n* ≥ 3). Values within the same row with different superscript letters are significantly different (*p* < 0.05).

^1^IBW, initial body weight (g).

^2^FR, feeding rate (%BW/day) = 100 × dry feed intake/(days × [initial body weight + final body weight + dead fish body weight]/2).

^3^FBW, final body weight (g).

^4^WGR, weight gain rate (%) = 100 × (final body weight − initial body weight)/initial body weight.

^5^SGR, specific growth rate (%/day) = 100 × (ln [final body weight] − ln [initial body weight])/days.

^6^SR, survival rate (%) = 100 × final fish number/initial fish number.

^7^FE, feed efficiency (%) = 100 × (final body weight − initial body weight)/dry feed intake.

^8^PRE, protein retention efficiency (%) = 100 × retained body protein/dietary protein intake.

^9^CF, condition factor (g/cm^3^) = 100 × body weight/(body length)^3^.

^10^VSI, viscerosomatic index = 100 × viscera weight/body weight.

^11^HSI, hepatosomatic index = 100 × liver weight/body weight.

**Table 3:** Liver biochemical parameters of a preponderant amphitriploid *Carassius* clone fed diets with different protein levels.

Items	P21	P24	P27	P30	P33	P36	P39
ALT (U/gprot)	12.06 ± 0.41^a^	11.58 ± 0.66^a^	12.80 ± 1.08^ab^	13.14 ± 0.26^ab^	13.89 ± 0.80^abc^	15.72 ± 0.56^c^	14.78 ± 1.21^bc^
AST (U/gprot)	14.93 ± 0.70	14.94 ± 0.79	14.68 ± 0.29	14.20 ± 0.23	13.58 ± 0.55	13.64 ± 0.77	13.28 ± 1.01
CAT (U/mgprot)	76.29 ± 4.55^b^	75.68 ± 3.10^b^	77.13 ± 4.21^b^	81.85 ± 3.66^b^	74.05 ± 3.60^b^	65.19 ± 6.55^ab^	51.47 ± 7.36^a^
MDA (nmol/mgprot)	1.27 ± 0.17^a^	1.21 ± 0.22^a^	1.44 ± 0.21^a^	1.75 ± 0.74^ab^	3.39 ± 0.62^ab^	3.77 ± 1.07^b^	3.89 ± 1.17^b^
HSP70 (ng/mL)	0.50 ± 0.22	0.78 ± 0.09	0.46 ± 0.15	0.99 ± 0.13	0.50 ± 0.15	0.77 ± 0.24	0.51 ± 0.14

*Note:* Data are presented as the mean ± SEM (*n* ≥ 3). Values within the same row with different superscript letters are significantly different (*p* < 0.05).

Abbreviations: ALT, alanine aminotransferase; AST, aspartate aminotransferase; CAT, catalase; HSP70, heat shock protein 70; MDA, malondialdehyde.

**Table 4:** Plasma biochemical parameters of a preponderant amphitriploid *Carassius* clone fed diets with different protein levels.

Parameters	P21	P24	P27	P30	P33	P36	P39
TP (g/L)	3.77 ± 0.12	3.88 ± 0.05	3.89 ± 0.08	3.68 ± 0.10	3.70 ± 0.06	3.96 ± 0.16	3.75 ± 0.12
GLU (mmol/L)	7.95 ± 1.42	8.58 ± 1.51	9.24 ± 0.65	9.42 ± 0.81	9.86 ± 0.76	10.55 ± 1.00	10.72 ± 0.71
TG (mmol/L)	3.72 ± 0.54^c^	3.46 ± 0.13^bc^	3.25 ± 0.29^abc^	2.85 ± 0.26^abc^	2.93 ± 0.36^abc^	2.72 ± 0.40^ab^	2.30 ± 0.18^a^
TC (mmol/L)	9.79 ± 0.48^d^	9.88 ± 0.25^d^	8.89 ± 0.36^cd^	8.34 ± 0.25^bc^	7.77 ± 0.45^ab^	7.04 ± 0.29^a^	6.78 ± 0.13^a^
HDL-c (mmol/L)	4.42 ± 0.24^c^	4.38 ± 0.17^c^	4.24 ± 0.15^c^	4.16 ± 0.14^c^	4.02 ± 0.14^bc^	3.69 ± 0.11^ab^	3.52 ± 0.07^a^
LDL-c (mmol/L)	2.43 ± 0.25^c^	2.21 ± 0.10^bc^	2.02 ± 0.13^b^	1.83 ± 0.09^ab^	1.61 ± 0.15^a^	1.53 ± 0.08^a^	1.46 ± 0.07^a^
ALT (U/L)	36.75 ± 8.45	38.48 ± 4.95	38.65 ± 4.39	31.94 ± 5.83	35.07 ± 4.45	34.80 ± 4.12	40.40 ± 9.25
AST (U/L)	173.73 ± 2.91	179.10 ± 5.82	186.40 ± 5.54	164.00 ± 2.12	178.60 ± 19.61	184.60 ± 16.26	170.67 ± 7.87
Ammonia (μmol/L)	241.62 ± 11.07^a^	241.11 ± 3.83^a^	254.94 ± 7.76^ab^	256.86 ± 13.77^ab^	265.02 ± 7.33^ab^	284.95 ± 16.65^b^	281.82 ± 17.07^b^
Cortisol (μg/L)	7.26 ± 0.96^a^	7.02 ± 0.67^a^	7.98 ± 1.39^ab^	9.83 ± 1.18^abc^	11.26 ± 1.06^bc^	12.44 ± 1.94^c^	11.87 ± 1.02^c^

*Note:* Data are presented as the mean ± SEM (*n* ≥ 3). Values within the same row with different superscript letters are significantly different (*p* < 0.05).

Abbreviations: ALT, alanine aminotransferase; AST, aspartate aminotransferase; GLU, glucose; HDL-c, high-density lipoprotein cholesterol; LDL-c, low-density lipoprotein cholesterol; TC, total cholesterol; TG, total triglyceride; TP, total protein.

**Table 5:** Free flavor amino acid contents (mg/g in fresh weight) in the muscle of a preponderant amphitriploid *Carassius* clone fed diets with different protein levels.

Items	P21	P24	P27	P30	P33	P36	P39
*Σ*Umami AA^1^	0.30 ± 0.05^b^	0.28 ± 0.06^b^	0.17 ± 0.01^ab^	0.12 ± 0.01^a^	0.16 ± 0.05^ab^	0.09 ± 0.02^a^	0.11 ± 0.03^a^
*Σ*Sweet AA^2^	3.50 ± 0.29^bc^	4.37 ± 0.28^c^	3.68 ± 0.17^bc^	3.86 ± 0.37^bc^	3.11 ± 0.40^ab^	2.55 ± 0.17^a^	2.95 ± 0.23^ab^
*Σ*Bitter AA^3^	16.46 ± 1.34^c^	17.01 ± 1.21^c^	15.67 ± 0.47^bc^	13.96 ± 0.15^bc^	14.70 ± 1.43^bc^	13.14 ± 0.68^ab^	10.85 ± 0.67^a^

*Note:* Data are presented as the mean ± SEM (*n* = 3). Values within the same row with different superscript letters are significantly different (*p* < 0.05).

^1^ *Σ*Umani AA, sum of umami amino acids (aspartic acid and glutamic acid).

^2^ *Σ*Sweet AA, sum of sweet amino acids (aspartic acid, glycine, alanine, methionine, leucine, arginine, and proline).

^3^ *Σ*Bitter AA, sum of bitter amino acids (valine, isoleucine, leucine, tyrosine, phenylalanine, histidine, and lysine).

**Table 6:** Main fatty acid composition (% of total fatty acids) in the muscle of a preponderant amphitriploid *Carassius* clone fed diets with different protein levels.

Items	P21	P24	P27	P30	P33	P36	P39
C14:0	5.10 ± 0.02^a^	5.16 ± 0.04^ab^	5.27 ± 0.04^bc^	5.19 ± 0.06^ab^	5.27 ± 0.03^bc^	5.35 ± 0.02^c^	5.36 ± 0.07^c^
C15:0	0.64 ± 0.01	0.65 ± 0.01	0.71 ± 0.02	0.72 ± 0.01	0.74 ± 0.00	0.79 ± 0.03	0.81 ± 0.01
C16:0	36.65 ± 0.09	37.10 ± 0.20	37.47 ± 0.21	37.47 ± 0.47	36.98 ± 0.24	36.75 ± 0.18	36.60 ± 0.36
C17:0	0.47 ± 0.00^a^	0.45 ± 0.02^a^	0.48 ± 0.00^ab^	0.51 ± 0.03^ab^	0.53 ± 0.00^bc^	0.58 ± 0.02^cd^	0.60 ± 0.02^d^
C18:0	7.93 ± 0.12^b^	7.07 ± 0.16^a^	7.61 ± 0.09^ab^	7.24 ± 0.35^ab^	7.28 ± 0.18^ab^	7.15 ± 0.30^ab^	7.40 ± 0.29^ab^
C20:0	0.14 ± 0.01^a^	0.14 ± 0.00^ab^	0.15 ± 0.00^ab^	0.14 ± 0.02^ab^	0.15 ± 0.00^ab^	0.16 ± 0.00^b^	0.15 ± 0.01^ab^
*Σ*SFA	50.93 ± 0.22^ab^	50.56 ± 0.03^a^	51.70 ± 0.26^c^	51.27 ± 0.31^bc^	50.95 ± 0.10^ab^	50.79 ± 0.27^ab^	50.92 ± 0.06^ab^
C16:1n-7	3.16 ± 0.06	3.31 ± 0.02	3.24 ± 0.05	3.32 ± 0.13	3.25 ± 0.03	3.22 ± 0.19	3.11 ± 0.13
C17:1n-7	0.30 ± 0.01^a^	0.31 ± 0.01^a^	0.33 ± 0.01^ab^	0.37 ± 0.01^cd^	0.36 ± 0.00^bc^	0.40 ± 0.01^d^	0.44 ± 0.02^e^
C18:1n-9	21.38 ± 0.38	21.95 ± 0.20	21.27 ± 0.30	21.94 ± 0.24	21.64 ± 0.07	21.42 ± 0.08	21.58 ± 0.28
C20:1n-9	1.31 ± 0.01	1.34 ± 0.05	1.20 ± 0.07	1.33 ± 0.01	1.29 ± 0.08	1.21 ± 0.08	1.24 ± 0.09
*Σ*MUFA	26.15 ± 0.44^ab^	26.92 ± 0.20^ab^	26.04 ± 0.33^a^	26.97 ± 0.19^b^	26.53 ± 0.09^ab^	26.25 ± 0.12^ab^	26.37 ± 0.33^ab^
C18:2n-6	12.16 ± 0.12^bcd^	11.90 ± 0.04^abc^	11.89 ± 0.13^ab^	11.60 ± 0.18^a^	12.20 ± 0.04^bcd^	12.50 ± 0.13^d^	12.34 ± 0.19^cd^
C18:3n-3	1.52 ± 0.02^ab^	1.52 ± 0.01^ab^	1.49 ± 0.04^ab^	1.43 ± 0.05^a^	1.50 ± 0.01^ab^	1.54 ± 0.03^b^	1.50 ± 0.02^ab^
C18:3n-6	0.11 ± 0.01	0.09 ± 0.01	0.13 ± 0.01	0.09 ± 0.01	0.10 ± 0.00	0.10 ± 0.01	0.11 ± 0.02
C20:2n-6	0.20 ± 0.01^ab^	0.18 ± 0.01^a^	0.19 ± 0.00^a^	0.20 ± 0.01^ab^	0.21 ± 0.00^b^	0.23 ± 0.01^c^	0.23 ± 0.01^c^
C20:3n-6	0.35 ± 0.01^ab^	0.34 ± 0.01^a^	0.34 ± 0.01^ab^	0.35 ± 0.01^ab^	0.36 ± 0.01^ab^	0.38 ± 0.02^b^	0.37 ± 0.01^ab^
C20:4n-6 (ARA)	0.40 ± 0.01	0.38 ± 0.02	0.42 ± 0.01	0.40 ± 0.02	0.40 ± 0.01	0.40 ± 0.03	0.39 ± 0.01
C20:5n-3 (EPA)	1.75 ± 0.03^b^	1.73 ± 0.05^b^	1.59 ± 0.02^a^	1.57 ± 0.08^a^	1.52 ± 0.02^a^	1.50 ± 0.05^a^	1.45 ± 0.02^a^
C22:2n-6	0.83 ± 0.01	0.82 ± 0.02	0.75 ± 0.01	0.74 ± 0.03	0.71 ± 0.01	0.70 ± 0.02	0.68 ± 0.01
C22:6n-3 (DHA)	5.09 ± 0.07	5.05 ± 0.20	4.85 ± 0.06	4.77 ± 0.17	4.91 ± 0.04	4.98 ± 0.08	4.93 ± 0.18
*Σ*n-3PUFA	8.35 ± 0.12	8.30 ± 0.23	7.93 ± 0.10	7.77 ± 0.27	7.93 ± 0.06	8.02 ± 0.14	7.87 ± 0.21
*Σ*n-6PUFA	14.04 ± 0.14^bc^	13.72 ± 0.01^ab^	13.71 ± 0.13^ab^	13.38 ± 0.23^a^	13.99 ± 0.04^bc^	14.31 ± 0.13^c^	14.12 ± 0.21^bc^
*Σ*HUFA	7.58 ± 0.11	7.50 ± 0.26	7.21 ± 0.08	7.09 ± 0.24	7.19 ± 0.05	7.26 ± 0.16	7.13 ± 0.22

*Note:* Data are presented as the mean ± SEM (*n* = 3). Values within the same row with different superscript letters are significantly different (*p* < 0.05).

Abbreviations: ΣHUFA, sum of highly polyunsaturated fatty acids; ΣMUFA, sum of monounsaturated fatty acids; Σn-3PUFA, sum of n-3 polyunsaturated fatty acids; Σn-6PUFA, sum of n-6 polyunsaturated fatty acids; ΣSFA, sum of saturated fatty acids.

**Table 7:** Whole body and muscle composition of a preponderant amphitriploid *Carassius* clone fed diets with different protein levels.

Parameters	P21	P24	P27	P30	P33	P36	P39
Whole body (% in fresh weight)							
Moisture	66.90 ± 0.32^ab^	65.72 ± 0.37^a^	67.14 ± 0.30^ab^	68.17 ± 0.83^bc^	68.76 ± 0.35^c^	68.71 ± 0.32^c^	68.98 ± 0.69^c^
Crude protein	15.20 ± 0.18^a^	15.56 ± 0.12^ab^	16.12 ± 0.03^bcd^	15.81 ± 0.10^b^	16.02 ± 0.27^bc^	16.64 ± 0.27^d^	16.52 ± 0.17^cd^
Crude lipid	12.05 ± 0.30^d^	12.51 ± 0.25^d^	10.93 ± 0.16^c^	10.62 ± 0.69^bc^	10.13 ± 0.14^abc^	9.48 ± 0.13^a^	9.75 ± 0.30^ab^
Ash	3.43 ± 0.02	3.56 ± 0.09	3.50 ± 0.07	3.26 ± 0.13	3.32 ± 0.07	3.36 ± 0.05	3.35 ± 0.13
Dorsal muscle (% in fresh weight)							
Moisture	73.18 ± 0.54^a^	74.52 ± 0.19^b^	75.53 ± 0.01^c^	75.39 ± 0.31^c^	75.87 ± 0.27^c^	75.89 ± 0.12^c^	76.20 ± 0.17^c^
Crude protein	17.63 ± 0.33^a^	17.95 ± 0.23^ab^	18.13 ± 0.38^ab^	18.20 ± 0.17^ab^	18.53 ± 0.30^ab^	18.73 ± 0.33^b^	18.58 ± 0.24^ab^
Crude lipid	8.51 ± 0.74^c^	6.66 ± 0.33^b^	5.27 ± 0.33^a^	5.44 ± 0.38^ab^	5.08 ± 0.14^a^	4.57 ± 0.53^a^	4.58 ± 0.21^a^

*Note:* Data are presented as the mean ± SEM (*n* = 3). Values within the same row with different superscript letters are significantly different (*p* < 0.05).

**Table 8:** Activities of digestive enzymes in the intestine of a preponderant amphitriploid *Carassius* clone fed diets with different protein levels.

Enzymes	P21	P24	P27	P30	P33	P36	P39
Trypsin (U/gprot)	97.77 ± 39.36^a^	106.53 ± 37.40^a^	119.08 ± 24.81^a^	136.06 ± 22.02^ab^	170.09 ± 33.85^ab^	185.37 ± 21.33^ab^	221.70 ± 25.57^b^
Chymotrypsin (U/gprot)	154.05 ± 39.42^a^	189.21 ± 45.17^a^	214.46 ± 35.33^a^	236.29 ± 59.40^a^	290.04 ± 45.94^ab^	390.90 ± 77.71^b^	437.56 ± 49.78^b^
Lipase (U/gprot)	30.33 ± 5.79	35.14 ± 7.87	35.00 ± 4.70	33.36 ± 5.38	19.67 ± 7.09	19.47 ± 5.74	29.36 ± 5.66
α-Amylase (U/mgprot)	0.93 ± 0.12	0.89 ± 0.08	0.96 ± 0.13	0.95 ± 0.08	1.15 ± 0.23	0.92 ± 0.19	1.00 ± 0.11

*Note:* Data are presented as the mean ± SEM (*n* ≥ 3). Values within the same row with different superscript letters are significantly different (*p* < 0.05).

## Data Availability

The data that support the findings of this study are available from the corresponding author upon reasonable request.

## Supporting Information

Supporting Information 1 Animal handling and monitoring procedures.docx: the information of animal handling and monitoring procedures during acclimatization and experimental trial.

Supporting Information 2 Completed author checklist—Full.pdf: the information of author contribution.

## References

[1] FAO (2024). *The State of World Fisheries and Aquaculture 2024—Blue Transformation in Action*.

[2] Lovell R. T. (1991). Nutrition of Aquaculture Species. *Journal of Animal Science*.

[3] McGoogan B. B., Gatlin D. M. (1999). Dietary Manipulations Affecting Growth and Nitrogenous Waste Production of Red Drum, *Sciaenops ocellatus* I. Effects of Dietary Protein and Energy Levels. *Aquaculture*.

[4] Quang Tran H., Van Doan H., Stejskal V. (2022). Environmental Consequences of Using Insect Meal as an Ingredient in Aquafeeds: A Systematic View. *Reviews in Aquaculture*.

[5] Mai K., Xue M., He G., Xie S. Q., Kaushik S. J., Hardy R. W., Kaushik F. E. (2022). Protein and Amino Acids. *Fish Nutrition*.

[6] Bušelić I., Trumbić Ž., Hrabar J. (2025). Unravelling the Intricate Language of Fish Guts: Impact of Plant-Based vs. Plant-Insect-Poultry-Based Diets on Intestinal Pathways in European Seabass. *Aquaculture*.

[7] Wang J., Chen L., Xu J. (2023). C1 Gas Protein: A Potential Protein Substitute for Advancing Aquaculture Sustainability. *Reviews in Aquaculture*.

[8] Kumar M., Tomar M., Punia S. (2021). Cottonseed: A Sustainable Contributor to Global Protein Requirements. *Trends in Food Science & Technology*.

[9] Islam S. M. M., Siddik M. A. B., Sørensen M. (2024). Insect Meal in Aquafeeds: A Sustainable Path to Enhanced Mucosal Immunity in Fish. *Fish & Shellfish Immunology*.

[10] Huangfu Y., Qu P., Liu D. (2024). Protein Requirements of Large Yellow Croaker *Larimichthys crocea* Depends on Protein Sources From the Perspective of Growth Performance, Digestive and Absorptive Enzyme Activities, Intestinal and Liver Histology. *Aquaculture Reports*.

[11] Tefal E., Peñaranda D. S., Martínez-Llorens S. (2024). Feeding of Rainbow Trout (*Oncorhynchus mykiss*) With Organic Ingredients Replacing Fish Meal. *Aquaculture*.

[12] Qu Y., Han F., Qiao Y., Shi X., Chen H., Li E. (2025). Effects of Replacing Soybean Meal With Fermented Rapeseed Meal in Low-Fish-Meal Feed on the Growth, Immunity, and Gut Microbiota of Juvenile White Shrimp, *Litopenaeus vannamei*. *Aquaculture*.

[13] Austreng E., Refstie T. (1979). Effect of Varying Dietary Protein Level in Different Families of Rainbow Trout. *Aquaculture*.

[14] Santos A. I., Nguyen N. H., Ponzoni R. W., Yee H. Y., Hamzah A., Ribeiro R. P. (2014). Growth and Survival Rate of Three Genetic Groups Fed 28% and 34% Protein Diets. *Aquaculture Research*.

[15] Zhou F., Ding X., Feng H. (2013). The Dietary Protein Requirement of a New Japanese Strain of Juvenile Chinese Soft Shell Turtle, *Pelodiscus sinensis*. *Aquaculture*.

[16] Ye W., Han D., Zhu X., Yang Y., Jin J., Xie S. (2017). Comparative Study on Dietary Protein Requirements for Juvenile and Pre-Adult Gibel Carp (*Carassius auratus gibelio* Var. CAS III). *Aquaculture Nutrition*.

[17] Xie S., Han D., Jin J., Xie S., Wang Z. (2023). Nutrition and Feed for Crucian Carp. *Conservation and Utilization of Crucian Carp Seed Resources*.

[18] Lee S., Small B. C., Patro B., Overturf K., Hardy R. W. (2020). The Dietary Lysine Requirement for Optimum Protein Retention Differs With Rainbow Trout (*Oncorhynchus mykiss* Walbaum) Strain. *Aquaculture*.

[19] Bureau of Fisheries of Ministry of Agriculture and Rural Affairs of the P.R.China (2024). National Fisheries Technology Extension Center, and China Society of Fisheries. *China Fishery Statistical Yearbook 2024*.

[20] Lu M., Zhou L., Gui J.-F. (2024). Evolutionary Mechanisms and Practical Significance of Reproductive Success and Clonal Diversity in Unisexual Vertebrate Polyploids. *Science China Life Sciences*.

[21] Chen Y., Zhu C., Zong Q., Yu B. (1986). Study on the Biology of White Crucian Carp. *Journal of Fisheries of China*.

[22] Ling Z. (1998). Artificial Breeding and Feeding Techniques of White Crucian Carp. *Fishery Guide to be Rich*.

[23] Luo L., Liu S., Ou Z., Yuan Z. (2021). Effect of Carbohydrate Level in Feed on Growth, Antioxidant Capacity and Muscle Composition of Crucian Carp. *Feed Research*.

[24] Lin K., Shen W., Zhuang Z. (1990). Comparisons on the Protein Digestibiliies of Feather Meals and Fish Meal in, *Carassius cuvieri* T.etS. *Journal of Huazhong Agricultural University*.

[25] AOAC (2012). *Official Methods of Analysis of the Association of Official Analytical Chemists*.

[26] Xu D., He G., Mai K., Zhou H., Xu W., Song F. (2016). Postprandial Nutrient-Sensing and Metabolic Responses After Partial Dietary Fishmeal Replacement by Soyabean Meal in Turbot (*Scophthalmus maximus* L.). *British Journal of Nutrition*.

[27] Fei S., Liu C., Xia Y. (2020). The Effects of Dietary Linolenic Acid to Linoleic Acid Ratio on Growth Performance, Tissues Fatty Acid Profile and Sex Steroid Hormone Synthesis of Yellow Catfish *Pelteobagrus fulvidraco*. *Aquaculture Reports*.

[28] Yu Y., Wang X., Jin J. (2024). Effects of the Replacement of Dietary Fishmeal by the Blend of *Tenebrio molitor* Meal, *Chlorella* Meal, *Clostridium Autoethanogenum* Protein, and Cottonseed Protein Concentrate on Growth, Protein Utilization, and Intestinal Health of Gibel Carp (*Carassius gibelio*, CAS Ⅴ). *Aquaculture Nutrition*.

[29] Taj S., Han Q., Wu X. (2023). Effects of Dietary Protein-to-Energy Ratios on Growth, Immune Response, Antioxidative Capacity, Liver and Intestinal Histology, and Growth-Related Gene Expression in Hybrid Yellow Catfish (*Pelteobagrus fulvidraco* ♀ × *Pelteobagrus vachelli* ♂). *Aquaculture Nutrition*.

[30] Zhang Y., Liang X., Zhan W. (2022). Effects of Dietary Protein Levels on Growth Performance, Muscle Composition and Fiber Recruitment of Juvenile Small Yellow Croaker (*Larimichthys polyactis*). *Aquaculture Reports*.

[31] Song M. Q., Yu Q. R., Li E. C. (2024). Leucine Improves Dietary Protein use Efficiency by Regulating Protein Synthesis by Activating Amino Acid Transporters and the mTORC1 Pathways in Chinese Mitten Crab (*Eriocheir sinensis*). *Aquaculture*.

[32] Liu T., Weng X., Wang J., Han T., Wang Y., Chai X. (2023). Dietary Protein Requirement of Juvenile Dotted Gizzard Shad *Konosirus punctatus* Based on the Variation of Fish Meal. *Animals*.

[33] Tu Y., Xie S., Han D. (2015). Growth Performance, Digestive Enzyme, Transaminase and GH-IGF-I Axis Gene Responsiveness to Different Dietary Protein Levels in Broodstock Allogenogynetic Gibel Carp (*Carassius auratus gibelio*) CAS III. *Aquaculture*.

[34] Gui J. F., Zhou L., Wang Z. W. (2018). Gibel Carp (*Carassius gibelio* Var. CAS V). *China Fisheries*.

[35] Duan C., Shimeno S., Hosokawa H., Takeda M. (1989). Protein Requirement of Fingerling Wuchan Fish, *Megalobrama amblycephala*. *Research Reports of the Kochi University Agricultural Science*.

[36] Li X.-F., Liu W.-B., Jiang Y.-Y., Zhu H., Ge X.-P. (2010). Effects of Dietary Protein and Lipid Levels in Practical Diets on Growth Performance and Body Composition of Blunt Snout Bream (*Megalobrama amblycephala*) Fingerlings. *Aquaculture*.

[37] Sabaut J. J., Luquet P. (1973). Nutritional Requirements of the Gilthead Bream *Chrysophrys aurata*. Quantitative Protein Requirements. *Marine Biology*.

[38] Singha K. P., Shamna N., Sahu N. P. (2020). Feeding Graded Levels of Protein to Genetically Improved Farmed Tilapia (GIFT) Juveniles Reared in Inland Saline Water: Effects on Growth and Gene Expression of IGF I, IGF-IR and IGF-BPI. *Aquaculture*.

[39] Grisdale-Helland B., Shearer K. D., Gatlin D. M., Helland S. J. (2008). Effects of Dietary Protein and Lipid Levels on Growth, Protein Digestibility, Feed Utilization and Body Composition of Atlantic Cod (*Gadus morhua*). *Aquaculture*.

[40] Yang S.-D., Liou C.-H., Liu F.-G. (2002). Effects of Dietary Protein Level on Growth Performance, Carcass Composition and Ammonia Excretion in Juvenile Silver Perch (*Bidyanus bidyanus*). *Aquaculture*.

[41] Walton M. J., Cowey C. B. (1977). Aspects of Ammoniogenesis in Rainbow Trout, *Salmo gairdneri*. *Comparative Biochemistry and Physiology Part B: Comparative Biochemistry*.

[42] Metón I., Mediavilla D., Caseras A., Cantó E., Fernández F., Baanante I. V. (1999). Effect of Diet Composition and Ration Size on Key Enzyme Activities of Glycolysis-Gluconeogenesis, the Pentose Phosphate Pathway and Amino Acid Metabolism in Liver of Gilthead Sea Bream (*Sparus aurata*). *British Journal of Nutrition*.

[43] Kirchner S., Kaushik S., Panserat S. (2003). Low Protein Intake Is Associated With Reduced Hepatic Gluconeogenic Enzyme Expression in Rainbow Trout (*Oncorhynchus mykiss*). *The Journal of Nutrition*.

[44] Fynn-Aikins K., Hughes S. G., Vandenberg G. W. (1995). Protein Retention and Liver Aminotransferase Activities in Atlantic Salmon Fed Diets Containing Different Energy Sources. *Comparative Biochemistry and Physiology Part A: Physiology*.

[45] Cai Y., Wermerskirchen J., Adelman I. R. (1996). Communications: Ammonia Excretion Rate Indicates Dietary Protein Adequacy for Fish. *The Progressive Fish-Culturist*.

[46] Brunty J. L., Bucklin R. A., Davis J., Baird C. D., Nordstedt R. A. (1997). The Influence of Feed Protein Intake on Tilapia Ammonia Production. *Aquacultural Engineering*.

[47] Jiang W. D., Deng Y. P., Zhou X. Q. (2017). Towards the Modulation of Oxidative Damage, Apoptosis and Tight Junction Protein in Response to Dietary Leucine Deficiency: A Likely Cause of ROS-Induced Gill Structural Integrity Impairment. *Fish & Shellfish Immunology*.

[48] Zhao W., Yao R., Wei H. L. (2023). Astaxanthin, Bile Acid and Chlorogenic Acid Attenuated the Negative Effects of High-Fat Diet on the Growth, Lipid Deposition, and Liver Health of *Oncorhynchus mykiss*. *Aquaculture*.

[49] Wang S., Tian J., Jiang X. (2023). Effects of Different Dietary Protein Levels on the Growth Performance, Physicochemical Indexes, Quality, and Molecular Expression of Yellow River Carp (*Cyprinus carpio haematopterus*). *Animals*.

[50] Qu F., Liu Z., Hu Y. (2019). Effects of Dietary Glutamine Supplementation on Growth Performance, Antioxidant Status and Intestinal Function in Juvenile Grass Carp (*Ctenopharyngodon idella*). *Aquaculture Nutrition*.

[51] Zhang Q., Chen Y., Xu W., Zhang Y. (2021). Effects of Dietary Carbohydrate Level on Growth Performance, Innate Immunity, Antioxidant Ability and Hypoxia Resistant of Brook Trout *Salvelinus fontinalis*. *Aquaculture Nutrition*.

[52] Yin P., Xie S., Zhuang Z. (2021). Chlorogenic Acid Improves Health in Juvenile Largemouth Bass (*Micropterus salmoides*) Fed High-Fat Diets: Involvement of Lipid Metabolism, Antioxidant Ability, Inflammatory Response, and Intestinal Integrity. *Aquaculture*.

[53] Wen C., Ma S., Tian H. (2022). Evaluation of the Protein-Sparing Effects of Carbohydrates in the Diet of the Crayfish, *Procambarus clarkii*. *Aquaculture*.

[54] Leppa S., Sistonen L. (1997). Heat Shock Response—Pathophysiological Implications. *Annals of Medicine*.

[55] Barton B. A. (2002). Stress in Fishes: A Diversity of Responses With Particular Reference to Changes in Circulating Corticosteroids. *Integrative and Comparative Biology*.

[56] S. M., Pailan G. H., Sardar P. (2023). Dietary Protein Requirement of Female Climbing Perch, *Anabas testudineus* (Bloch, 1792) Broodstock. *Animal Feed Science and Technology*.

[57] Liao Z., Liu Y., Wei H. (2023). Effects of Dietary Supplementation of, *Bacillus subtilis*, DSM 32315 on Growth, Immune Response and Acute Ammonia Stress Tolerance of Nile Tilapia (*Oreochromis niloticus*) Fed With High or Low Protein Diets. *Animal Nutrition*.

[58] Liu C., Liu H., Zhu X. (2022). The Effects of Dietary *Arthrospira platensis* on Oxidative Stress Response and Pigmentation in Yellow Catfish *Pelteobagrus fulvidraco*. *Antioxidants*.

[59] Ma X. Z., Feng L., Wu P. (2020). Enhancement of Flavor and Healthcare Substances, Mouthfeel Parameters and Collagen Synthesis in the Muscle of on-Growing Grass Carp (*Ctenopharyngodon idella*) Fed With Graded Levels of Glutamine. *Aquaculture*.

[60] Xu J., Feng L., Jiang W. D. (2018). Different Dietary Protein Levels Affect Flesh Quality, Fatty Acids and Alter Gene Expression of Nrf2-Mediated Antioxidant Enzymes in the Muscle of Grass Carp (*Ctenopharyngodon idella*). *Aquaculture*.

[61] Dong M., Zhang L., Wu P. (2022). Dietary Protein Levels Changed the Hardness of Muscle by Acting on Muscle Fiber Growth and the Metabolism of Collagen in Sub-Adult Grass Carp (*Ctenopharyngodon idella*). *Journal of Animal Science and Biotechnology*.

[62] Rahimnejad S., Dabrowski K., Izquierdo M., Malinovskyi O., Kolářová J., Policar T. (2021). Effects of Dietary Protein and Lipid Levels on Growth, Body Composition, Blood Biochemistry, Antioxidant Capacity and Ammonia Excretion of European Grayling (*Thymallus thymallus*). *Frontiers in Marine Science*.

[63] Gao Y., Lu S., Wu M., Yao W., Jin Z., Wu X. (2019). Effects of Dietary Protein Levels on Growth, Feed Utilization and Expression of Growth Related Genes of Juvenile Giant Grouper (*Epinephelus lanceolatus*). *Aquaculture*.

[64] Yan X., Yang J., Dong X. (2021). Optimum Protein Requirement of Juvenile Orange-Spotted Grouper (*Epinephelus coioides*). *Scientific Reports*.

[65] Wang J., Jiang Y., Li X. (2016). Dietary Protein Requirement of Juvenile Red Spotted Grouper (*Epinephelus akaara*). *Aquaculture*.

[66] Giri S. S., Sahoo S. K., Sahu A. K., Meher P. K. (2003). Effect of Dietary Protein Level on Growth, Survival, Feed Utilisation and Body Composition of Hybrid Clarias Catfish (*Clarias batrachus* × *Clarias gariepinus*). *Animal Feed Science and Technology*.

[67] Ye C., Wu Y., Sun Z., Wang A. (2017). Dietary Protein Requirement of Juvenile Obscure Puffer, *Takifugu obscurus*. *Aquaculture Research*.

[68] Guo W., Fu L., Wu Y. (2021). Effects of Dietary Protein Levels on Growth and Feed Utilization in Non-Transgenic and Growth-Hormone-Gene Transgenic Common Carp (*Cyprinus carpio* L.). *Aquaculture Reports*.

[69] Fazio F. (2019). Fish Hematology Analysis as an Important Tool of Aquaculture: A Review. *Aquaculture*.

[70] Xv Z.-C., He G.-L., Wang X.-L., Shun H., Chen Y.-J., Lin S.-M. (2021). Mulberry Leaf Powder Ameliorate High Starch-Induced Hepatic Oxidative Stress and Inflammation in Fish Model. *Animal Feed Science and Technology*.

[71] Wang J., Lan K., Wu G. (2022). Effect of Dietary Carbohydrate Level on Growth, Feed Utilization, Energy Retention, Body Composition, and Digestive and Metabolic Enzyme Activities of Juvenile Cobia, *Rachycentron canadum*. *Aquaculture Reports*.

[72] Barlaya G., Ananada Kumar B. S., Hegde G., Kannur H. (2021). Nutrient Digestibility and Digestive Enzyme Activity in Fringe Lipped Carp, *Labeo fimbriatus* (Bloch, 1795), Fed Diets Containing Cottonseed Meal. *Asian Fisheries Science*.

[73] Willora F. P., Vatsos I. N., Mallioris P. (2022). Replacement of Fishmeal With Plant Protein in the Diets of Juvenile Lumpfish (*Cyclopterus lumpus*, L. 1758): Effects on Digestive Enzymes and Microscopic Structure of the Digestive Tract. *Aquaculture*.

[74] Qian J., Xiao L., Feng K. (2022). Effect of Dietary Protein Levels on the Growth, Enzyme Activity, and Immunological Status of *Culter mongolicus* Fingerlings. *PLoS ONE*.

[75] Toledo-Solís F. J., Martínez-García R., Galaviz M. A., Hilerio-Ruiz A. G., Álvarez-González C. A., de Rodrigáñez M. S. (2020). Protein and Lipid Requirements of Three-Spot Cichlid *Cichlasoma trimaculatum* Larvae. *Fish Physiology and Biochemistry*.

