# Supplementary material for: A New Strain of Preponderant Amphitriploid Carassius Clone Juvenile With Integrated Genomes Partly From White Crucian Carp (C. auratus cuvieri) Requires Low Dietary Protein
Source: Aquac Nutr. 2025 Jan 22;2025:6356786. doi: 10.1155/anu/6356786 (PMC11779906; doi:10.1155/anu/6356786)
Supplement: Supporting Information 1 — Animal handling and monitoring procedures.docx: the information of animal handling and monitoring procedures during acclimatization and experimental trial. [file 6356786.f1.docx]

**1. Fish collection**

Before the growth trial, all fish was cultured in an outdoor pond of the Breeding Center of Crucian Carp of the Ministry of Agriculture and Rural Affairs of the P. R. China (Liangzi Lake Research Base, the Institute of Hydrobiology, Chinese Academy of Sciences, Wuhan, Hubei, P. R. China). They were cautiously captured by a trawling, and gently transferred to the rearing system in Institute of Hydrobiology, Chinese Academy of Sciences in the container with oxygen aeration by car (about 1 hour trip).

**2. Fish acclimatization**

After arrival, fish in the container were carefully transferred into an indoor recirculating water system (gross water volume: 30 m^3^) and hand-fed with a commercial diet twice a day. Water quality was controlled at an acceptable level for the fish. This acclimatization lasted for 2 weeks.

**3. Growth trial**

After the acclimatization, feeding was stopped for 24 hours to empty the gut. Then 420 similar size and apparent healthy and active fish were randomly selected and equally released into 21 cylindrical fiberglass tanks (water volume: 285 L, water depth: 0.58 m). Every selection was gently conducted by hands and the fish were kept to minimum stress. The 21 tanks were randomly divided into seven groups for the seven experimental diets. During the 8-week growth trial, fish were hand-fed to apparent satiation at 8:30, 13:30 and 16:30 and the uneaten feed were collected 0.5 h after each meal. Dead fish were promptly removed and recorded. The water temperature was recorded every morning and afternoon, which ranged within 26.4 ± 5.1°C. The pH value, dissolved oxygen and ammonia nitrogen concentrations were determined every fortnight. They were controlled at 7.44 ± 0.13, more than 6.08 mg/L and less than 0.15 mg/L, respectively. Light density was 100~120 lx near the water surface, and residual chlorine was less than 0.02 mg/L.

**4. Sample collection**

After the growth trial, fish were again fasted for 24 hours, then fish in each tank were anaesthetized by MS-222 (tricaine methanesulfonate, Argent Chemical Laboratories Inc., Redmond, WA, USA), gently scooped out by a fishing net and bulk weighed. Eight fish in each tank were randomly selected, of which two were weighed and stored at −20 °C for determining the whole body composition, amino acids and fatty acid profiles. Three fish were conducted to caudal blood sampling using 2 mL heparinized syringes, then these three fish were dissected on ice, and livers, midguts and dorsal muscles were immediately detached for succedent assay. After the body lengths were measured, another three fish were weighed, dissected, and their viscera and livers were isolated and weighed, for the calculation of morphological parameters.
